# Life-threatening complications of high doses of intravenous methylprednisolone for treatment of Graves’ orbitopathy

**DOI:** 10.1186/s13044-019-0074-0

**Published:** 2019-12-23

**Authors:** Dorota Walasik-Szemplińska, Grzegorz Kamiński, Iwona Sudoł-Szopińska

**Affiliations:** 1 Ophthalmic Hospital “Sensor Cliniq”, Kacza 8, 01-013, Warsaw, Poland; 2 Department of Endocrinology and Radioisotope Therapy, Military Institute of Medicine, Warsaw, Poland; 3 Department of Radiology, National Institute of Geriatrics, Rheumatology and Rehabilitation, Warsaw, Poland; 4 Department of Medical Imaging, Second Faculty of Medicine, Medical University of Warsaw, Warsaw, Poland

**Keywords:** Methylprednisolone, Graves’ disease, Graves’ Orbitopathy

## Abstract

**Background:**

Treatment of moderate to severe Graves’ orbitopathy (GO) is based mainly on intravenous pulses of methylprednisolone. High doses of methylprednisolone can exert several adverse effects, some of which might be life-threatening. The objective of this study is to describe the most severe complications associated with intravenous administration of high doses of glucocorticoids, and to develop the patient examination standards prior to their qualification for the therapy.

**Main body:**

In this paper, we describe the most severe, life-threatening complications of intravenous methylprednisolone and address their possible underlying mechanism. We also present recommendations and precautions which should be taken prior to initiation of intravenous pulses of methylprednisolone treatment for GO. To address risk of hepatic complications, we recommend regular monitoring of biochemical parameters of hepatic function. Additionally, assessment of the risk of cardiovascular events should be undertaken based on medical history, estimation of risk factors, and investigations, such as determination of thyroid hormones and thyroid-stimulating hormone levels, electrolyte and glucose concentrations, electrocardiogram examination and measurements of blood pressure.

**Conclusions:**

An individualized safe and effective dose of intravenous methylprednisolone should be established for each patient with GO based on the vascular risk factors, comorbidities, and concomitant drugs. According to the European Group on Graves’ Orbitopathy (EUGOGO) guidelines, cumulative doses of intravenous methylprednisolone should not exceed 8 g.

## Background

Graves’ orbitopathy (GO, also referred to as ophthalmopathy) is a chronic autoimmune disease that manifests as orbital cellulitis and is presently observed in 25–50% of patients with Graves’ disease [1]. Efficient treatment for this disease has been a constant challenge for the last two centuries with regard to the selection of therapy as well as the time when the treatment can be initiated. The selection of an appropriate treatment protocol is based on disease activity. Severity, progression in time, age, presence of coexisting medical disorders, and the existence of risk factors for the occurrence or exacerbation of orbitopathy can influence the decision about the beginning of the therapy and the dose of intravenous methylprednisolone. One of the criteria for selecting a suitable therapy includes foreseeing the possible side effects and estimating the risk of their occurrence individually for each patient. It is important to note that treatment of GO is a complex procedure, consisting of both restoration and maintenance of the euthyroid state, elimination of risk factors, and providing treatment aimed at restricting the inflammation of the orbit.

Since the 1950s, glucocorticoids have been used in the treatment of GO. Three different routes of drug administration are known: topical (in the form of peri- or retrobulbar and subconjunctival injections, [2–4] oral, and intravenous. The steroids, independently of their route of administration, function by limiting the action of both B and T lymphocytes, restricting the influx of monocytes and macrophages, and reducing the activity of immunocompetent cells, the secretion of inflammatory mediators (prostaglandins and cytokines) and the synthesis of glycosaminoglycans. The combined effects of all the above-mentioned actions also diminish the activity of orbital fibroblasts [5].

Undoubtedly, intravenous administration of glucocorticoids is the first-line therapy for the serious form of GO, in particular when there is a risk for optic neuropathy [6]. They can also be used to treat moderate and occasionally mild forms of the disease which manifest with intensified external symptoms. The risk of spontaneous progression from mild to moderate and further to severe form is comparatively low, being observed only in 15–16% of the patients in the randomized controlled studies [7, 8]. However, identification of the sub-group of patients with a high risk of disease progression remains difficult, and systemic therapy is recommended based on the presence of the following prognostic factors: smoking, high serum thyroid stimulating hormone receptor antibody (TRAb) concentration, and presence of traits that are reflective of the active form of the disease. However, many authors do not advocate systemic treatment with glucocorticoids in the case of mild GO owing to the risk of severe systemic complications [9], Table 1.

**Table 1:** Occurrence of complications of glucocorticoid therapy in patients with Graves’ orbitopathy (based on Marcocci et al., 2001 [9])

	Oral (*n* = 27)	Intravenous (*n* = 41)
Mild to moderate:		
Hyperglycemia, gain in body weight, depression, and Cushingoid features	17	27
Severe:		
Cardiovascular, cerebrovascular, > 4-fold increase in liver enzymes, acute liver failure, autoimmune hepatitis	10	14

*n* number of physicians reporting adverse events

The intravenous route of methylprednisolone has been highly recommended for GO, considering the highest efficacy of the therapy and low number of associated complications [9–13]. Currently, topical administration of glucocorticoid is used for GO only occasionally, where there is a high risk of dangerous complications with systemic administration. The limitation of this route includes unfavorable adverse effects induced by triamcinolone acetate, including increase in orbital pressure with an increase in the dosage of the drug, need for multiple injections, and extensive scarring of tissues [3, 14]. The use of steroids has been proven to be effective in reducing the edema and consolidating the muscles, which in turn result in reduced fibrogenesis. The impact of steroid therapy on proptosis remains restricted [15].

Despite the wide application of glucocorticoids in the treatment of autoimmune diseases, determination of their safety profile is the key in planning the treatment for GO. Various randomized controlled trials have confirmed higher efficacy and lower number of adverse events with intravenous pulses of glucocorticoids in comparison with oral steroid therapy [11, 13, 16]. Therefore, the intravenous administration route has gained a wide acceptance and comprises the first-line therapy for moderate and severe forms of GO. However, reports of associated severe side effects should prompt the development of patient examination standards before implementation of therapy with intravenous pulses of methylprednisolone.

The initiation of the systemic glucocorticoid therapy should be based on the general and topical status of the patient and the assessment of the risk factors for disease progression. The treatment regimen should meet the expectations of the patient and be preceded by a discussion, wherein the patient is informed about the risks that can arise from the proposed therapy. In addition, the physician should present in detail the benefits as well as the possible side effects that can be encountered during the therapy. The objective of this review is to describe and discuss the most severe complications that may occur following intravenous administration of high doses of glucocorticoids, and to develop patient examination standards prior to their qualification for the therapy.

## Main text

### Complications of systemic steroid therapy

Owing to the emergence of severe adverse events following intravenous administration of methylprednisolone pulses during the treatment of active GO form, a group of researchers, members of the European Thyroid Association and the European Group on Graves’ Orbitopathy (EUGOGO), in 2012 performed a quantitative estimation of the rate of complications of this therapy [9]., Table 1 Marcocci et al. conducted a survey by sending the questionnaire to 349 experienced clinicians, members of the European Thyroid Association, including questions concerning adverse events that had occurred during or after glucocorticoid therapy in patients with GO [9]., Table 1 Data obtained from 148 respondents treating patients with GO enabled analysis of the incidence rate of adverse events in patients undergoing glucocorticoid therapy.

The data sent by 128 clinicians, who confirmed treating GO with glucocorticoids at their centers, showed that 65% used intravenous route of administration while 25% used oral route. The remaining clinicians (10%) used other therapeutic modalities, i.e., orbital radiotherapy.

The complications associated with glucocorticoid therapy in patients with GO are listed in Table 1 and Table 2.

**Table 2:** Severe complications of intravenous glucocorticoid therapy in patients with Graves’ orbitopathy

Organ system	Report	Complication	Dose of methyl-prednisolone
Hepatic complications	Marino et al. [17]	Acute liver failure (7 out of 800 patients, including 3 lethal cases)	3.0–24.0 g
	Weissel and Hauff [18]	Acute liver failure (lethal, 1 patient)	15.0 g
	Salvi et al. [19]	Acute liver failure (1 patient)	5.5 g
Cardiovascular complications	Owecki et al. [20]	Severe hypertension leading to myocardial infarction (1 patient)	5.0 g
	Gursoy et al. [21]	Severe hypertension leading to acute hear failure without myocardial infarction (1 patient)	2.0 g
	Lendorf et al. [22]	Myocardial infarction (1 out of 49 patients) Angina pectoris (2 out of 49 patients) Ischemic stroke (lethal, 1 out of 49 patients) Pulmonary embolism (lethal, 1 out of 49 patients)	2.0–5.0 g

### Incidence of complications associated with oral steroid therapy

About 81% of the responders from the EUGOGO survey reported to have observed side effects during treatment with oral steroid therapy [9], Table 1. The adverse events were qualified as severe in 10 cases, including cardiovascular (*n* = 1) and cerebrovascular/cerebral (*n* = 3) events. A fourfold increase in the serum concentration of liver enzymes was also observed following systemic steroid therapy (*n* = 6). Two patients died during treatment due to cerebrovascular complications. The total dosage of glucocorticoids in both cases was 2.3 g of prednisone. One patient was diagnosed with severe congestive failure, and pulmonary embolus occurred in another case. In 17 patients, adverse effects were classified as moderately severe. The most commonly reported side effects in this group of patients included hyperglycemia, increase in body weight, depression, and Cushingoid features.

### Incidence of complications associated with intravenous steroid therapy

In the group of patients treated intravenously, a total of 32 from 83 clinicians (39%) reported 53 adverse effects, of which 27 qualified as severe complications: cardiovascular (*n* = 10), cerebrovascular (*n* = 5), acute liver failure (*n* = 3), autoimmune encephalitis (*n* = 1), and over fourfold increase in the level of liver enzyme concentrations (*n* = 8) [9], Table 1. The others were mild to moderate (*n* = 14) [9], Table 1. Due to the emergence of severe complications following the immunosuppressive treatment, a total of seven female patients died, due to acute liver failure (*n* = 4), and cardiovascular (*n* = 1) and cerebrovascular (*n* = 2) complications; three of them deceased during the therapy, and four of them after completion of the therapy. The total dose of methylprednisolone in the deceased patients was 8–15 g. The most commonly reported adverse side effect was an acute increase in the level of liver enzymes (fourfold higher than the normal value), and this condition is often asymptomatic.

#### Hepatic toxicity

Hepatic toxicity of intravenous glucocorticoids has been described by numerous clinicians [Table 2]. This complication is rare, but it may cause irreversible liver injury and may ultimately lead to death.

Marino et al. reported on seven cases of acute liver failure, which occurred in 800 patients treated with intravenous methylprednisolone, of which three cases resulted in death [17]., Table 2 Early traits of liver disease were not evident in any of these patients. The symptoms appeared during the treatment in two of the three deceased patients, while it occurred after the completion of therapy in one patient. The onset of symptoms (jaundice, lassitude, anorexia, and diarrhea) was sudden, and blood tests revealed a considerable (over fourfold higher than the normal level) increase in the levels of liver enzymes and bilirubin. Immunosuppressive therapy was immediately interrupted. However, this did not prevent the development of cascade of events and a rapid deterioration of the general status and biochemical parameters, resulting in the death of two patients within 7 weeks; nevertheless, liver transplant was performed in the third patient who died later as a result of kidney complications. Furthermore, an asymptomatic increase of liver enzymes was observed during therapy in four patients, which led to immediate halting of the therapy. Their biochemical parameters spontaneously returned to normal values within 9–22 weeks [17], Table 2.

Weissel and Hauff reported acute liver failure in a Caucasian woman aged 71 years, who underwent intravenous pulses of methylprednisolone treatment for severe active GO [18], Table 2. Four weeks after the completion of the treatment, a rapid increase in the concentration of liver enzymes and alterations in the synthesis of clotting factors were observed. Despite intensive treatment, the patient died within 10 days. Post-mortem histopathological examination revealed necrosis of the liver parenchyma. Serological and immunohistochemical tests were negative for the presence of hepatitis A, B, and C antigens [18], Table 2.

Salvi et al. reported the case of a 43-year-old woman who developed acute liver failure following treatment with pulses of methylprednisolone for GO associated with Hashimoto’s disease [19], Table 2 After completion of the treatment, a rapid increase in serum aminotransferase concentration was observed (1200 U/L). Serology tests for the presence of hepatitis A, B, and C antigens were negative. Before the influence of the applied therapy, the biochemical parameters were within normal limits. Four weeks after the completion of intravenous therapy, a liver biopsy demonstrated autoimmune hepatitis (AIH) [19], Table 2.

By analyzing the aforementioned cases, one should contemplate whether alternate treatment regimens for methylprednisolone intravenous pulses exist, which can decrease the incidence of severe adverse reactions, and whether side effects depend on the total dosage of the administered drug. An analysis of 23 studies of patients with GO who received intravenous glucocorticoids showed that nearly all severe events occurred with cumulative doses of methylprednisolone greater than 8 g; therefore, the cumulative dose of this drug should not exceed 8 g [5].

It needs to be highlighted that the treatment regimens that resulted in acute liver failure had been previously applied in numerous patients, and that drug dosages did not vary significantly from the widely approved treatment regimens.

In the group of seven patients described by Marino et al. [17], different treatment regimens were used over the years, where patients were administered 7.5–15 mg/kg daily for 8–16 weeks. The total dosage ranged between 3 and 24 g. The total dosage in patients who died due to acute liver failure was found to be slightly higher (10.8 ± 3.6 g) than in those who recovered (7.9 ± 2.9 g). However, this difference was not statistically significant.

In the case reported by Weissel and Hauff [18], Table 2, the patient was treated with five cycles of 1 g methylprednisolone pulses, wherein each cycle involved administration of the drug for 3 consecutive days. After a pulse cycle, oral treatment with prednisone at a dosage decreasing to 0 was carried out in the period of 10–14 days. The cycle intervals were between 14 days and 6 weeks. The total dose used in this case was 15 g, and as underlined by the authors, the dosage regime is widely used for treating patients with multiple sclerosis.

In the case reported by Salvi et al. [19], Table 2, methylprednisolone was administered at a dosage of 7.5 g/cycle for every 2 weeks, and each cycle involves administration of the drug for 2 consecutive days. The total dose was relatively low (5.5 g).

A positive association between the total dose of methylprednisolone and an increased risk of hepatic complications was demonstrated in two studies. Le Moli et al. compared two groups of patients treated with intravenous methylprednisolone for GO [23]. In the first group comprising 13 patients, the total dose of administered methylprednisolone was 8.45 g, while in the second group comprising 14 patients the dosage was 4.5 g. Increased concentrations of liver enzymes were observed in 38% of patients in the first group and in 14% of patients in the second group. However, only in 7 out of 13 patients from both groups (5 in the first group and 2 in the second group), the enzyme levels increased over the normal values [23].

A study by Zang et al., which compared various treatment regimens involving different dosages of intravenous methylprednisolone pulses, demonstrated a strong association between the total drug dose and the incidence of acute liver failure [5]. Lethal liver damage was observed in cases where high cumulative dosages of methylprednisolone, between 10 and 24 g, were used [5].

The possible causes for the above-mentioned adverse effects remain unknown. First and foremost, glucocorticoids can directly affect the liver cells, similar to the effect exhibited by hepatotoxic drugs. Mitochondrial damage and alterations in the beta-oxidation of fatty acids occur, leading to the release of free oxygen radicals and depletion of ATP stock in the liver cells. As an effect, steatosis occurs, which can be confirmed by the following findings: a positive correlation between the occurrence of acute liver failure and dosage, the onset of manifested symptoms during therapy rather than after its completion, earlier consumption of potentially hepatotoxic drugs. In five out of seven patients preexisting liver steatosis was demonstrated in ultrasonography in the Marino report [17], Table 2.

The second probable mechanism of liver injury is through immunological suppression caused by glucocorticoids. Based on this hypothesis, it can be assumed that virus-induced hepatitis can occur in the liver cells. In one patient treated with low dose methylprednisolone, and in whom lethal acute liver failure occurred, a high concentration of anti-cytomegalovirus (anti-CMV) IgM was recorded. An additional finding supporting this hypothesis is that the alanine transaminase (ALT) / aspartate transaminase (AST) ratio was < 1 in all described cases. However, it should be emphasized that hepatitis B virus (HBV) and CMV viruses were not observed in five out of seven patients, and tests for determining the presence of herpes simplex virus (HSV) and hepatitis C virus (HCV) were also negative [17, 19].Table 2.

The third mechanism by which glucocorticoid use leads to hepatotoxicity may be via a “rebound” mechanism. Following the administration of glucocorticoids, a suppression of the immune system is observed, and sudden discontinuation of glucocorticoids may lead to autoaggression and result in AIH. The following findings support this hypothesis: in three out of seven patients, acute liver failure occurred after completion of treatment (4.9 ± 1.2 weeks), and positive test for the presence of *liver-kidney microsomal* (LKM) antibodies, which is associated with lymphocytic consolidation in one patient and the presence of antinuclear autoantibodies in another patient. It should also be emphasized that of the seven patients with acute liver failure discussed earlier, none received maintenance dose of the steroid either between methylprednisolone pulses or after completion of the therapy, which may have prevented the occurrence of adverse events through this mechanism [17, 19, 20], Table 2.

The study of Marino et al. [17], Table 2 showed that out of 800 patients acute hepatic failure occurred in 0.8% of the cases, with lethal outcome observed in 0.3% of the patients. However, it should be noted that asymptomatic increase in the enzyme levels occurs more frequently, particularly after completion of the treatment, but is usually not reported due to the lack of a standardized biomarker that can reflect the hepatic parameters accurately after the completion of the treatment.

According to Le Moli et al., the asymptomatic increase in the level of liver enzymes (AST, ALT) also depends on the total dosage of methylprednisolone [23]. Increase of the enzyme level is associated with increased bilirubin or gamma-glutamyl-transpeptidase (GGTP) levels, but the concentration of alkaline phosphatase is reduced, which stems from the inhibiting effect of steroids on osteoclasts. The increased enzyme level is typically observed after the first administration of methylprednisolone, and according to authors, steroid administration-dependent liver injury does not correlate with the preceding hepatic steatosis or concomitant diabetes [23].

Moreover, one cannot ignore the fact that increase of aminotransferases may also stem from the hepatotoxic effect of antithyroid drugs or thyrotoxicosis itself [9].

According to a research study [9], a maximum dosage of 8 g is relatively safe for the proper functioning of the liver, with the condition that its functioning is regularly examined.

#### Vascular complications

Apart from the reports on acute liver failure and increased serum concentrations of hepatic enzyme, exceeding normal values by several fold, there are papers documenting cerebrovascular and cardiovascular disorders, the incidence of which is closely related to the time of administration of methylprednisolone pulses to treat patients with active GO.

The occurrence of several cases of myocardial necrosis and acute coronary syndromes were documented in patients, without preceding cardiac disorders, following treatment with methylprednisolone intravenous pulses. Owecki et al. [22], Table 2 described the case of a 66-year-old woman, who was treated with methylprednisolone pulses according to the following protocol: 1 g of methylprednisolone/day for 3 subsequent days, and then after an interval of 4 days, another 3-day cycle was repeated. Apart from receiving treatment for hypothyroidism, which occurred after therapy with radioiodine, the patient did not take any other drug. This was her second course of intravenous therapy with methylprednisolone. The previous treatment has been given 18 months earlier. The second treatment was administered 4 months after the return of symptoms of active GO. In the medical history of the patient, there was a coronary episode, 2 years earlier, of a transient coronary ischemia, but despite the diagnosis, the treatment was not given. The patient smoked cigarettes, about 10/day.

On the second day, during the fifth dose of the second cycle methylprednisolone administration, a rapid elevation of blood pressure occurred (190/110 mmHg). The symptoms of acute ischemia and myocardial necrosis were observed, which was further confirmed by elevated cardiac necrosis markers (CPK-MB- isoenzyme MB of creatine phosphokinase was 319 IU/l and troponin T level exceeded 2 ng/ml (reference range < 0.1 ng/ml), electrocardiography (ECG), and echocardiography [22], Table 2.

Similar case was reported by Gursoy et al. [23], Table 2 regarding a 53-year-old healthy man who was treated with methylprednisolone for moderate form of GO. Symptoms of GO appeared 6 months after the treatment with radioiodine. The scheme of administration of glucocorticoids was as follows: 1 g of methylprednisolone/day for 2 subsequent days, every 2 weeks. On the second day, after administration of the infusion, a sudden increase of blood pressure (180/120 mmHg) and acute pulmonary edema (with unclear cause) were noticed. Echocardiogram (ECG) demonstrated a 35% reduction in ejection fraction and a decrease in volume loading of the left ventricle. Multiple repetitions of the ECG, normal values of cardiac enzymes, as well as cardiac catheterization excluded myocardial necrosis. The precise cause for the sudden change in the cardiopulmonary homeostasis remains unknown. However, it appears that the sudden expansion of the fluid in the vascular bed and a rapid elevation in the blood pressure induced by the action of glucocorticoids in combination with prolonged uncontrolled thyrotoxicosis resulted in acute pulmonary edema [23], Table 2.

In a study by Lendorf et al. [24], Table 2, a group of 49 patients were treated with methylprednisolone pulses for active GO. A total of 67 therapies were used, according to the following protocol: 1 g of methylprednisolone/day for 5 subsequent days. In five patients, severe vascular complications were observed: two patients died, one patient developed myocardial infarction, and acute coronary syndrome occurred in two patients. The first deceased patient was a white woman aged 24 years, who after thyroidectomy was taking L-thyroxine. The only risk factor associated with the development of vascular complications was tobacco smoking, approximately 15–30 cigarettes/day. Due to active GO, the patient was initially treated with oral prednisolone at a dosage of 30 mg/day, but the therapy was stopped due to depression and progression of Cushingoid features. Surgical orbital decompression was performed, which yielded good results. Subsequently, a 9-month therapy with cyclosporin A was given. After 3 months of the therapy, a sudden decrease in visual acuity and recrudescence of the activity of autoimmune process in the orbits occurred, and the patient was offered treatment with intravenous methylprednisolone pulses. During the fourth dose of methylprednisolone, the patient reported visual disturbances and emotional instability, which resolved spontaneously. On seventh day, 2 days after completion of the therapy, computed tomography (CT) scan of the head demonstrated ischemia in the right middle cerebral artery with intensified brain edema and right ventricular compression. Severely impaired levels of consciousness and periods of asystole occurred. Despite intensive treatment, brain edema increased, and midline shift was observed. The patient died 2 days later. Autopsy revealed thrombosis of the right middle cerebral artery without features of inflammation.

The second case of lethal complication following treatment with high doses of intravenous glucocorticoids occurred in a 71-year-old woman with paroxysmal atrial fibrillation, for which she was treated with digoxin and clopidogrel. Another risk factor for vascular complications was smoking cigarettes at 10/day. The patient had undergone thyroidectomy 30 years earlier. She was treated with intravenous methylprednisolone pulses for active GO, followed by orbital irradiation and cyclosporine A. Considering the reduced visual acuity and color vision disorders, it was decided to administer another cycle of intravenous pulse of glucocorticoids. On the third day of the therapy, after administration of 3 g of methylprednisolone, shortness of breath and atrial fibrillation occurred. Although treatment was continued, circulatory–respiratory failure occurred, and despite resuscitation the patient died. Autopsy revealed pulmonary embolus and thrombosis within coronary vessels.

The third patient, without prior cardiac problems, was treated on two occasions with methylprednisolone pulses within a span of 1 year. The total dose of glucocorticoid administered during the first therapy was 5 g, and in the second cycle, the dose was reduced to 500 mg of methylprednisolone/day for 5 consecutive days (a total of 2.5 g). On the second day of the therapy, patient reported chest pain and shortness of breath. ECG revealed left bundle branch block and ST elevations across the precordial leads. Myocardial infarction was confirmed by the presence of high levels of cardiac enzymes [24], Table 2.

In all the described cases, there was a close temporal correlation between the occurrence of vascular events and administration of intravenous glucocorticoids, hence it was assumed that the treatment was the direct cause of the adverse events.

The thyrotoxicosis status also has an impact on the hemodynamic cardiovascular parameters. Excessive production of thyroid hormones is associated with increased filling of the vascular bed, reduction of vascular tone, and positive inotropic and chronotropic effects, which results in shortened circulation time. Despite the enhancement of both contractility and relaxation of the myocardium, reduction of the cardiac reserve occurs, and this effect is reversed by antithyroid drugs. Left untreated, thyrotoxicosis in the long term may lead to myocardial infarction without concomitant cardiac abnormalities [24].

In the case of patients described by Owecki [22], Table 2 and Gursoy [23], Table 2, the authors assumed that rapid elevation in blood pressure levels destabilized the previously stable atherosclerotic coronary plaque, resulting in coronary artery occlusion. In addition, disturbance in the volumic balance and overuse of the left ventricle, which overlapped with hemodynamic changes resulting from endocrine disorders, could have led to pulmonary edema and circulatory–respiratory failure.

It is traditionally assumed that glucocorticoids induce sodium and fluid retention. They increase both systolic and diastolic pressure independently of the renin-angiotensin–aldosterone system. However, the precise mechanism remains unknown. The current research in this field focuses on the impact of glucocorticoids on blood vessels, mediated by nitric oxide. Increased contractility of arteries and increased vascular tone are observed as a result of the impact of glucocorticoids on both the endothelium and the smooth muscles of the vessel walls. In the endothelial cells, glucocorticoids inhibit the reduction of vasodilator factors, such as prostacyclins and nitric oxide. In the vascular smooth muscles, the effect of steroids is diverse, and it is regulated by 11 beta-hydroxysteroid dehydrogenase (11beta-SD) [25].

Patients, in whom lethal vascular complications occurred, did not have prior cardiac problems, which would have disqualified them from undergoing intravenous therapy with glucocorticoid pulses. However, tobacco smoking was the common risk factor, which besides having a negative impact on the occurrence and exacerbation of GO, and reduction in the therapeutic effect of glucocorticoids, could have also contributed to the occurrence of adverse vascular events.

The issue concerning total dosage and its possible correlation with adverse vascular events remains a topic of discussion. Lendorf et al. pointed out that severe vascular complications occurred after the administration of total methylprednisolone dose of 5–8 g, including the dosage of preceding treatment [24], Table 2 Owecki [22], Table 2 states that myocardial ischemia occurred in a patient during the administration of fifth dose of methylprednisolone (5 g), but previous treatment with this drug was not taken into account (two cycles of 3 g each, i.e., 6 g in total). These dosages do not exceed the generally used doses and those recommended by the EUGOGO; however, considerable discrepancies between total dosages should be emphasized.

In a study by Jastrzębska, a statistically significant increase in the percentage of side effects was demonstrated in patients undergoing repeated glucocorticoid therapy in comparison to those undergoing treatment course for the first time (59% vs 41%) [26]. However, it remains unknown whether a considerable gap should be maintained between glucocorticoid courses, so that the dose is not summed up and can be considered safe [26].

## Conclusions

Glucocorticoids are used in the treatment of numerous autoimmune diseases. Despite the published reports on complications arising from treatment with high doses of steroids, such as infections, arterial hypertension, increased blood glucose levels, neuropsychiatric complications, liver function abnormalities, glaucoma, sudden cardiac death due to ventricular arrhythmias, and myocardial infarction, it can be stated that the therapeutic value of the treatment may exceed the risk of their use. A multicenter, randomized, double-blind study compared the efficacy and safety of three cumulative doses of methylprednisolone (2.25 g, 4.98 g, and 7.47 g) given intravenously over 12 weeks to 159 patients with GO [27]. The highest dose caused the greatest improvement, but it was associated with an increased risk of major adverse events. Thus, the intermediate dose was recommended for most patients and the highest dose only for those with severe GO [27]. These recommendations were later endorsed by the EUGOGO [28].

It should also be emphasized that higher therapeutic effect following the coadministration of glucocorticoids and orbital radiation therapy has been demonstrated, which enables a reduction in the total dose of steroids, treatment time, and consequently a reduction in the incidence of adverse symptoms [29, 30]. However, the effect of orbital radiotherapy was investigated in patients who received oral glucocorticoids. There are no data on the effect of orbital radiation therapy in conjunction with intravenous glucocorticoids, although the combination of oral glucocorticoids with radiation therapy seems more effective than intravenous glucocorticoids alone [27].

Considering the reports describing severe complications after methylprednisolone treatment, regular monitoring of biochemical parameters of hepatic function is recommended. All patients should be tested regularly for liver enzyme concentrations, AST, ALT, GGT, bilirubin, C- reactive protein (CRP) and factors of the coagulation system. If the baseline levels exceed normal levels, it is a contraindication for administration of high doses of glucocorticoids. Prior to undergoing treatment, markers of hepatitis B and C should be determined. Some authors also recommend the evaluation of antibodies associated with AIH. The presence of hepatic steatosis and other abnormalities in liver ultrasonography should be taken into account as risk factors for the occurrence of serious complications during treatment with high doses of glucocorticoids. In the report by Marino et al. [17], Table 2, 2 out of 7 patients with acute liver damage had liver steatosis on abdominal ultrasound before the initiation of glucocorticoids. However, because liver steatosis is common (~ 33% of adults in the USA have this condition [31]), it would be impractical to disqualify all patients with any degree of liver steatosis from glucocorticoid treatment. Perhaps higher grades of liver steatosis can be considered as a contraindication for this treatment. Qayyum et al. [32] proposed a grading system for hepatosteatosis based on the fat content of hepatocytes: normal (< 5%), mild (5–33%), moderate (34–66%), and severe (> 66%). This system could be used for risk stratification among patients who receive glucocorticoids for GO.

Other factors should also be considered when assessing the risk of hepatotoxicity. For example, statins, commonly used cholesterol-lowering drugs, may increase the hepatotoxicity of glucocorticoids. Covelli et al. described two patients with GO in whom intravenous methylprednisolone caused liver damage, which resolved only after discontinuing statins [33].

Assessment of the risk of cardiovascular events should be based on medical history, estimation of risk factors, and additional tests. Prior to treatment with high doses of glucocorticoids, serum thyroid hormones and TSH levels, electrolytes, and glucose concentrations should be determined. ECG as well as blood pressure measurements should be performed.

It is probably impossible to determine a safe dose, whose therapeutic effect would be satisfactory. Keeping in mind the vascular risk factors, comorbidities, and concomitant drugs, a safe dose should be established for each patient individually. Unfortunately, despite the careful selection of appropriate dosage, no treatment is currently available whose side effects could be eliminated completely.

## Data Availability

Not applicable.

## Abbreviations

11beta-SD: 11 beta-hydroxysteroid dehydrogenase
AIH: Autoimmune hepatitis
ALT: Alanine transaminase
AST: Aspartate transaminase
CMV: Cytomegalovirus
CPK-MB: Isoenzyme MB of creatine phosphokin
CRP: C- reactive protein
CT: Computed tomography
ECG: Electrocardiography
EUGOGO: European Group on Graves’ Orbitopathy
GGTP: Gamma-glutamyl-transpeptidase
GO: Graves’ orbitopathy
HBV: Hepatitis B virus
HCV: Hepatitis C virus
HSV: Herpes simplex virus
LKM: Liver-kidney microsomal
rr: Relative risk
TRAb: Thyroid stimulating hormone receptor antibody
TSH: Thyroid-stimulating hormone
